# The Neural Mechanism of Encountering Misjudgment by the Justice System

**DOI:** 10.1371/journal.pone.0075434

**Published:** 2013-09-25

**Authors:** Qian Cui, Qinglin Zhang, Hidehiko Takahashi

**Affiliations:** 1 Faculty of Psychology, Southwest University, Chongqing, China; 2 Department of Psychiatry, Kyoto University Graduate School of Medicine, Kyoto, Japan; 3 Key Laboratory of Cognition and Personality (Southwest University), Ministry of Education, Chongqing, China; George Mason University/Krasnow Institute for Advanced Study, United States of America

## Abstract

Although misjudgment is an issue of primary concern to the justice system and public safety, the response to misjudgment by the human brain remains unclear. We used fMRI to record neural activity in participants that encountered four possible judgments by the justice system with two basic components: whether the judgment was right or wrong [accuracy: right vs. wrong (misjudgment)] and whether the judgment was positive or negative [valence: positive vs. negative]. As hypothesized, the rostral ACC specifically processes the accuracy of judgment, being more active for misjudgment than for right judgment, while the striatum was uniquely responsible for the valence of judgment, being recruited to a larger extent by positive judgment compared to negative judgment. Furthermore, the activity in the rACC for positive misjudgments was positively correlated with that for negative misjudgments, which confirmed the misjudgment-specificity of the rACC. These results demonstrate that the brain can distinguish a misjudgment from a right judgment and regard a misjudgment as an emotionally arousing stimulus, independent of whether it is positive or negative, while positive judgment is considered as hedonic information, regardless of whether it is right or wrong. Our study is the first to reveal the neural mechanism that underlies judgment processing. This mechanism may constitute the basis of future studies to develop a novel marker for the detection of lies.

## Introduction

A misjudgment is an inaccurate factual determination by the justice system, which produces harmful effects on the social security system, and especially, brings serious consequences to the individuals involved. As accuracy and fairness are the goals of law enforcement [1], misjudgments receive large amounts of publicity and have attracted a critical mass of studies by criminologists and other social scientists [2]. However, these studies have mostly focused on wrongful convictions and investigated the causes and consequences [3], [4] from the legal [5], [6], public [1], [7] and witness [8], [9] perspectives. To date, no research has focused on the mental and neural responses that occur when encountering misjudgment from the standpoint of the interrogee.

In order to obtain more accurate determinations of veracity, recent studies have been working to develop a reliable method to detect deception using fMRI technology, which can identify brain activity patterns associated with cognitive processes with modest temporal resolution and high spatial resolution. These studies found that the prefrontal-parietal and anterior cingulate cortices, as the executive brain regions, are pivotally involved in deception [10]–[17]. However, because of the nonspecific relationship between deception and these executive regions, sufficient accuracy has not been obtained with the use of these executive regions to discriminate the guilty from the innocent [18]. Many publications in this area have pointed out this disadvantage [19]–[21], and some researchers believe that it is difficult to find a simple biological marker that can indicate deception, as this is a highly complex and multifaceted cognitive process. A potential solution to this issue might not be to address deception in its totality, but to isolate more precise associated processes, in order to seek new markers of deception [22]. In this study, we not only wanted to investigate the unknown neural mechanism for encountering misjudgment, but we also aimed to seek a neural marker for whether the judgment coincides with the truth. This could be used to distinguish between right and wrong judgments, with the ultimate aim of detecting a lie.

In actual justice situations, misjudgments may result in two consequences: that of a guilty person going free and the conviction of an innocent person. From the point of view of the interrogee, the former is advantageous (positive misjudgment), while the latter is disadvantageous (negative misjudgment). To simulate this situation, a novel paradigm named the “Judgment Game” was used in this study to generate four possible outcomes that included two basic components of judgment: whether the judgment is right or wrong [accuracy: right judgment vs. wrong judgment (misjudgment)], and whether the judgment is advantageous or disadvantageous to the interrogee (valence: positive vs. negative). This design enables us to investigate the neural mechanisms that underlie processing the accuracy and valence of judgment from the perspective of the interrogee, and furthermore to test whether the misjudgment-related neural responses vary with differences in the misjudgment valence (positive vs. negative).

The accuracy of judgment describes whether the judgment is right or wrong. Misjudgment is actually a kind of error that is conducted by a third-party justice system. Previous electrophysiological, neuroimaging and lesion studies have consistently confirmed the critical role of ACC in error processing [23]–[30]. Furthermore, recent studies have shown that the ACC is also recruited while observing errors performed by others [31]–[33]. We thus hypothesized that misjudgment would also activate ACC involved in error processing, regardless of whether the misjudgment is positive or negative to the interrogee.

In contrast, the determining of valance of whether the judgment results in a reward or loss to the interrogee would recruit the cognitive and neural mechanisms of processing rewards. As the striatum has been consistently reported to be recruited by primary and social reward processing [34]–[39], we hypothesized that positive judgment, as a hedonic outcome, will activate the striatum to a larger extent compared with negative judgment, regardless of whether the judgment is right or wrong.

If these hypotheses are true, the accuracy and valence of judgment could be disassociated in the human brain.

## Methods

### Participants

Fifteen undergraduates were recruited as participants from Southwest University in China and paid after the experiment. One was excluded because of excessive head movement, and 14 subjects remained for the analysis (7 men and 7 women; mean age [±SD], 21.93±1.90 yr.; range, 19 to 26 yr.). All subjects were right-handed and free from any physiological or psychological disease. After the procedures were fully explained, all subjects provided informed written consent according to the Declaration of Helsinki (BMJ 1991; 320: 1194) [40]. This study was approved by the ethics committee of Southwest University of China.

### Procedure

The participant was asked to play a “Judgment Game” with another participant who was actually a confederate. Firstly, the experimenter simply introduced the rules: “The game simulates a court situation in which two suspects compete against each other to allege their innocence in a financial theft. One of the suspects is the actual thief. To identify the thief, the justice system uses a lie detection program, which can compare the brain signals of the two suspects. However, some people can control their brain signals during lying (e.g., professional spies) and are therefore better at telling lies than others, which might cause the computer to judge wrongly. One wins if he/she is judged as innocent regardless of whether this is true or not and therefore avoids any penalty”. Subsequently, the two players were asked to draw cards labeled with “red” or “blue”, representing the color of their own doors from which feedback would appear during the game. To facilitate data analysis, every participant was manipulated to choose the “red” card. After they learned the instructions of how to play the game and practiced for 20 rounds, the two players were brought into two separate rooms for scanning. Unknown to the participants, their co-players did not actually play the game.

**Figure 1:**
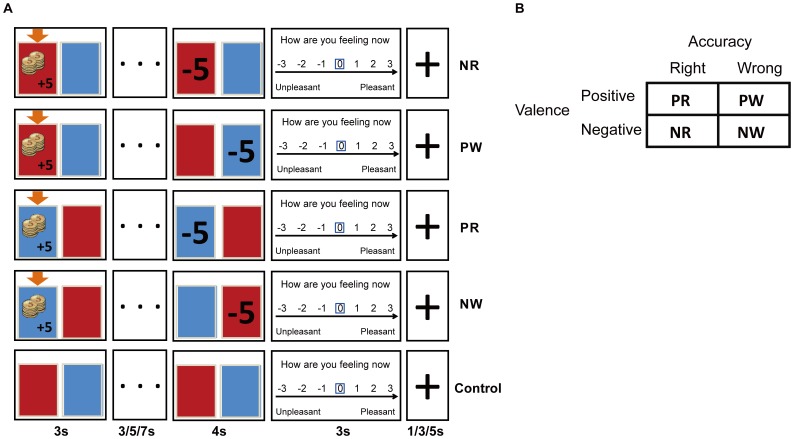
Schematic representation of the experimental procedure and design. (A) Four possible event sequences and the control event during a single trial of the judgment game. The orange arrow indicated which player was transferred ¥5 and was thus chosen to be the “thief”. An ellipsis appeared for a varied duration to represent the period when the computer analyzed brain signals from both players. The “−5” feedback appeared on one of two doors indicated who was judged as the “thief” and was penalized ¥5. Finally, a scale that ranged from −3 to 3 appeared on the screen and was used by participants to rate their subjective pleasure regarding the previous judgment. (B) The design matrix of this study: two basic components of judgment (accuracy and valence) with two levels of each were crossed to generate four kinds of judgment.

Each trial included following steps (Figure 1A): firstly, the participant was presented with an image of a “money exporter” and two doors for 3 s. The red door (participant’s door) would appear on the left or right side with an equal probability. The door located on the left side would show the transfer of 5 Yuan (¥, Chinese currency) and the gainer would thus be imaged as the “thief”. During this stage, the participant was instructed to answer which door (left or right) presenting on the screen was the thief’s door by pressing either the “1” or “4” keys on the keyboard with their left or right thumbs, respectively. As the participant should always “allege” that his/her co-player (blue door) was the “thief”, he/she should press “1” when the blue door appeared on the left or “4” when the blue door appeared at the right side. After making allegation, the participant was shown an ellipsis for a varied duration of 3, 5 or 7 s, which represented a “judging stage” during which the computer analyzed the brain signals of both players. Then, a feedback (judgment) stage occurred, during which a “−5” appeared either on the door of participant (red) or co-player (blue) for 4 s, informing who was judged as the “thief” and thus penalized by ¥5. Finally, a rating scale appeared on the screen, and the participants rated their feelings about the judgment on a 7-point scale that ranged from −3 (unpleasant) to 3 (pleasant) by moving a rectangular cursor along the scale with button presses. The rating had to be given within 3 s, followed by a varied inter-trial interval of 1, 3 or 5 s. The duration of each trial ranged from 14 to 22 s.

In reality, the judgments given by the computer were experimentally predetermined to be either consistent or inconsistent with the facts. Four types of trial were therefore generated: 1) money was transferred to the participant, who was then rightly judged as the thief (right judgment with a negative valence, NR); or 2) the co-player was wrongly judged as the thief (wrong judgment with a positive valence, PW; positive misjudgment); 3) money was transferred to the co-player, and the participant was wrongly judged to be the thief (wrong judgment with negative valence, NW; negative misjudgment); or 4) the co-player was rightly judged as the thief (right judgment with a positive valence, PR) (Figure 1B). In an additional control condition, no money was transferred, and therefore neither player was judged to be a criminal and penalized. The presentations of the stimulation in five types of trial are illustrated in Figure 1A.

If the participant responded incorrectly, instead of showing a judgment, a warning appeared that stated, “The red gave an incorrect response”, followed by a blank screen for 3 s as the equivalent of the rating stage. Additionally, eight filler trials were used to emphasize that the co-player was indeed playing the game. In these trials, the feedback given was always, “The blue gave an incorrect response”, independent of the response of the participant.

The formal experiment consisted of 158 trials in total, including 120 experimental trials (30 of each type), 30 control trials and eight filler trials. The trials were interspersed randomly with all trials being divided into five sessions of approximately 9 min each. Between two sessions, the participant could have a break for about 3 minutes. At the beginning of each session, to make it more credible that the participant was really playing against the co-player he/she met before, the experimenter called each player’s name separately and asked if they were ready for the next scanning. Then, the confederate who was actually sitting in the operation room would answer “I am ready” through the microphone, so that the participant could hear his/her voice.

At the end of the scans, one trial was randomly picked from the set, and the gain or loss for that trial was paid out. The primary fee for participation was ¥30. After the experiment, the participants would be asked with three questions: 1) if they believed that they were playing with the co-player; 2) if they believed that the judgments were given by the computer based on their brain signals; and 3) if they used any strategies during the game.

### fMRI Data Acquisition

Images were acquired using a 3T Siemens Magnetom TrioTim B17 MRI scanner equipped with a standard polarized head coil (Siemens Medical Systems, Erlangen, Germany). The T2*-weighted gradient echo planar imaging (EPI) sequence sensitive to blood oxygenation level dependent (BOLD) contrast was used to obtain the functional images of 1,476 volumes. Each volume included 32 axial and interleaved acquired 3-mm thick slices with 1-mm gap, being oriented parallel to the AC-PC plane (TR = 2,000 milliseconds; TE = 30 milliseconds; FOV = 220×220; matrix = 64×64; flip angle = 90°). High-resolution T1-weighted images composed of 176 volumes were also acquired for each participant as anatomical reference (TR = 1,900 milliseconds; TE = 2.52 milliseconds; slice thickness = 1 mm; FOV = 256×256; voxel size = 1×1×1 mm).

### fMRI Data Analysis

Data processing and analysis were performed using the Statistical Parametric Mapping software package (SPM8; Wellcome Department of Cognitive Neurology, London, UK) running with MATLAB (Mathworks, Natick, MA). Images were first-slice timing corrected and realigned to correct for head motion, and then spatially normalized based on the functional EPI template provided by SPM8. Images were smoothed using a Gaussian kernel with a full width at a half maximum of 8 mm, finally generating images of 2×2×2 mm^3^ cubic voxels.

The data were analyzed in an event-related manner. At the first (individual) level, a general linear model (GLM) was used to model each trial as a boxcar, and its onset corresponded to judgment onset with duration of 0, being convolved with a hemodynamic response function. We created separate regressors for each of the four conditions (accuracy: right vs. wrong; valence: positive vs. negative) for each subject. Six head-motion parameters from subject-specific realignment were also modeled as regressors of no interest to correct for movement-related artifacts, and a high-pass filter set at 128 sec was applied to the data to remove low frequency noise. Linear contrasts of regression coefficients were calculated at the individual subject level and were entered into a group-level random-effects analysis to estimate the error variance across individuals. At the second (group) level, a full factorial design was used with accuracy and valence as two within-subject factors. Whole-brain analyses were thresholded at false discovery rate (FDR) <0.05 combined with cluster level corrected, FWE <0.05.

Furthermore, to focus on the fMRI signals responding to misjudgment, based on the results from the main effect of accuracy, we created a functional region of interest (ROI), composed of all voxels exceeding the threshold at FDR <0.05 combined with cluster level corrected, FWE <0.05, generating just one ROI locating the rACC. Parameter estimates were extracted from this ROI (rACC) for each participant and each condition, averaged across all voxels, and were subsequently analyzed using a repeated-measures ANOVA with accuracy (right vs. wrong) and valence (positive vs. negative) as within-subject factors, in order to further confirm the misjudgment-specificity of this area (i.e. to test whether it also showed valence or accuracy by valence interaction effects).

Finally, to confirm our hypothesis that the same cognitive process underlay the rACC activations for both positive and negative misjudgments, a correlation analysis was performed to assess the relationship between the rACC activities for PW (positive misjudgment) and NW (negative misjudgment) to test the more direct links between these two types of misjudgment.

## Results

### Manipulation Check

All participants believed that they were playing against a counterpart during scanning and that judgments were formed by the computer according to their brain signals. Interestingly, some participants even discussed with their counterparts who was better at telling lies when they met again after scanning. No specific strategies were reported, with the exception that some participants mentioned that they tried to keep calm after money was transferred.

### Behavioral Data

**Figure 2:**
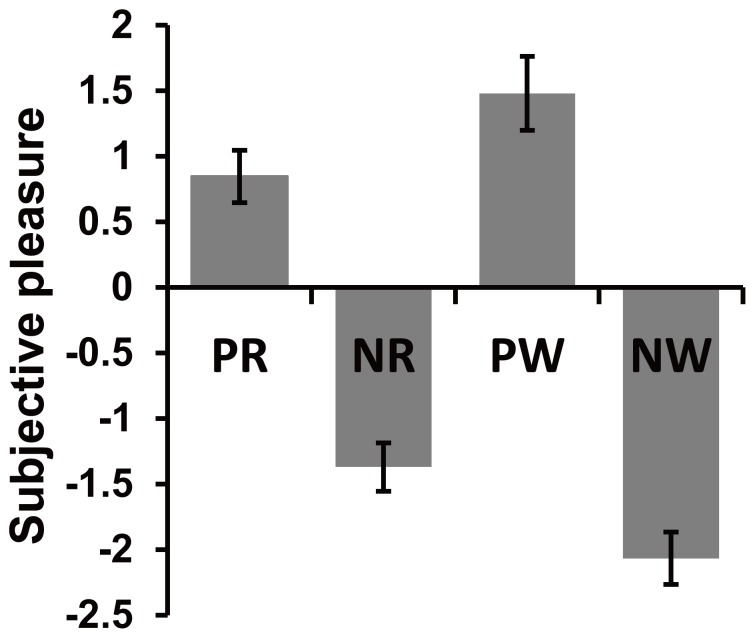
Behavioral results showing the effect of judgment manipulation. Subjective rating regarding the pleasure for four types of judgments (mean ± SEM) was modulated by the valence of judgment.

The average ratings of participants in the four types of trial are shown in Figure 2. A two (accuracy)×two (valence) repeated-measures ANOVA using the rating score as the dependent variable revealed a significant main effect of valence (*P = *0.000, partial eta squared [η_p_^2^] = 0.827). This suggested that the participants felt significantly more pleasure in ‘other-punished’ (mean = 1.164) than self-punished conditions (mean = −1.719), and there was no significant accuracy effect (*P = *0.804, η_p_^2^ = 0.005). Paired t-tests were conducted to compare the ratings in control conditions (neutral) against those for each of the four kinds of judgment. Both positive judgments (PR and PW) were ranked as being significantly more pleasant than the control condition (*t*_(13) = _4.629, *P = *0.000, cohen’d = 1.237 for PR and *t*_(13) = _5.256, *P* = 0.000, cohen’d = 1.405 for PW), while both negative judgments (NR and NW) were ranked as significantly more unpleasant than the control condition (*t*_(13) = _7.440, *P = *0.000, cohen’d = 1.988 for NR and *t*_(13) = _10.831, *P* = 0.000, cohen’d = 2.895 for NW). This finding confirmed the positive valence of other-punished judgments and the negative valence of self-punished judgments irrespective of whether the judgments were right or wrong. Furthermore, a significant accuracy by valence interaction effect was found (*P* = 0.000, η_p_^2^ = 0.662). Within other-punished conditions, participants felt more pleasant for wrong than right judgments (*P* = 0.007, η_p_^2^ = 0.436), while in self-punished conditions, the opposite result was found, as the ratings for right judgments were significant greater than those for wrong judgments (*P* = 0.001, η_p_^2^ = 0.587). In other words, participants had the most pleasant feelings with positive misjudgments and the most unpleasant were associated with negative misjudgments.

### fMRI Data

As the main interest of this study was to explore the accuracy and valence mechanisms in the brain during processing judgments, we focused on two general contrasts. The activated brain areas are summarized in Table 1.

**Table 1:** Brain regions showing significant activity by whole brain analyses.

Contrast	Activated region	BA	t	Cluster Size	x	y	z
**Wrong>right**	R rostral anterior cingulate	32	5.52	2889	6	42	14
**Positive>negative**	R striatum	–	7.32	1624	10	14	−2
	L postcentral gyrus	3/4	6.06	1121	−34	−22	50
	R lingual gyrus	18	5.56	504	12	−76	2
**Negative >positive**	R postcentral gyrus	3/4	7.95	1674	54	−14	52
	R posterior insula	13	5.79	616	44	−20	22
	L lingual gyrus	18	5.07	1061	−16	−54	−20

(FDR <0.05 combined with cluster level corrected, FWE <0.05).

Notes: x, y, z indicate the MNI coordinates of the local peak of each cluster. R, the right hemisphere; L, the left hemisphere. Neither the right>wrong contrast nor the interaction (accuracy×valence) showed any significant activations.

**Figure 3:**
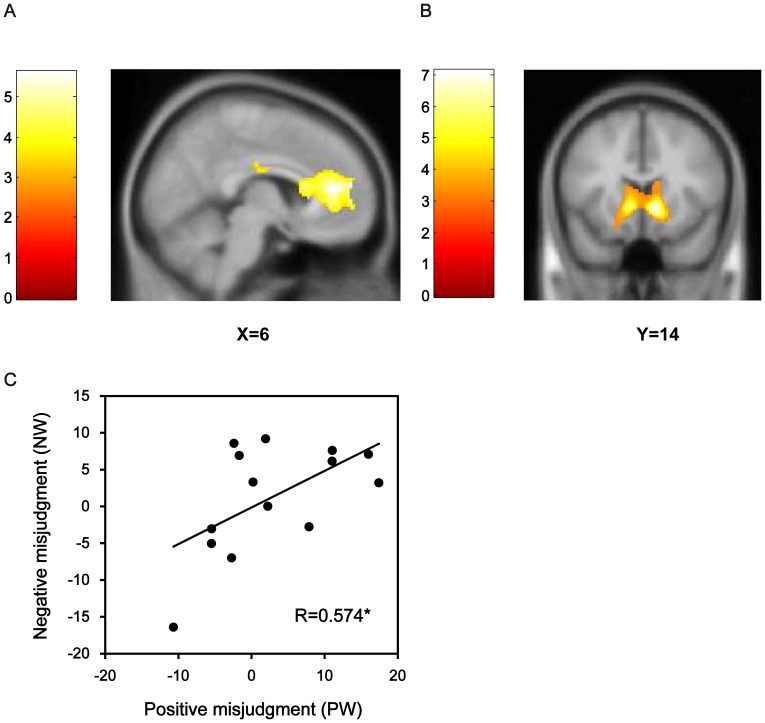
fMRI results showing misjudgment-specific and reward-specific brain activations. All maps are thresholded at FDR <0.05 combined with cluster level corrected, FWE <0.05. (A) Sagittal view of rACC activity (at MNI coordinates x = 6) increased in cases of right judgments compared to those with wrong judgments. (B) Coronal view of the striatum activity (at MNI coordinates y = 14) increased in cases of positive judgments compared to negative judgments. (C) Plots and regression line of correlation between rACC activations for positive misjudgment conditions (PW) and that for negative misjudgment conditions (NW) (*r* = 0.574, *P = *0.032).

As expected, misjudgment-specific activity was clustered in the ACC, especially in the rACC. Specifically, no brain areas were more active in response to right judgments than to wrong judgments. Wrong judgments, meanwhile, elicited more activations around the rACC than right judgments, including the right and left rACC and left medial frontal cortex (local maximum at x = 6, y = 42, z = 14; Figure 3A). Consistent with previous studies [32], [34], [36], [41] and our hypotheses, the valence-specific activations were mainly located in the striatum, with a maximum in the caudate, for positive judgment compared with negative judgment (including the right and left caudate; local maximum at x = 10, y = 14, z = −2; Figure 3B). In addition, the left postcentral gyrus and the right lingual gyrus were activated more strongly by positive judgments than by negative judgments, while the right posterior insula, the right postcentral gyrus and the left lingual gyrus showed greater activity levels with negative judgments than positive (Table 1). The interaction effect revealed no significant signal change.

When focusing on the functional ROI (rACC) defined by the activated areas associated with misjudgment, two (accuracy)×two (valence) ANOVAs yielded a significant main effect of accuracy (*F* (1, 13) = 60.028; *P = *0.000, η_p_^2^ = 0.822) and non-significant main effect of valence (*F* (1, 13) = 0.445; *P* = 0.516, η_p_^2^ = 0.033) or accuracy by valence interaction (*F* (1, 13) = 0.018; *P = *0.895, η_p_^2^ = 0.001). These results showed that activities in the rACC increased for both types of misjudgment, independent of whether it was advantageous or disadvantageous to the subject. Confirming this observation, a direct comparison between positive misjudgment (PW) and negative misjudgment (NW) using the paired t-test revealed no significant differences in the parameter estimates within rACC ROIs (*t*_(13)_ = 0.760, *P* = 0.461, cohen’d = 0.196). Correlation analysis revealed a significantly positive correlation in the degree of activations of the rACC (*R* = 0.574, *P* = 0.032) between positive and negative misjudgment conditions (Figure 3C).

## Discussion

This study demonstrated that the brain activations during encountering judgment from a third-party justice system showed two disassociated brain patterns for processing the accuracy and valence of the judgment, respectively. As predicted, wrong judgments produced greater activity in the ACC compared to right judgments, and the rACC was specifically activated, regardless of whether the judgment was positive or negative for the participant. However, the striatum was the main region responsible for processing the valence of judgment, being more active for positive than negative judgment, irrespective of whether the judgment was right or wrong. These results supported the hypothesis that, during judgment processing, the neural mechanism of processing judgment accuracy is fully disentangled from that involved in processing the valence. Furthermore, the rACC activities associated with positive misjudgment were significantly correlated with those associated with negative misjudgment, suggesting that the rACC played the same role in the processing of both types of misjudgment. This finding further confirmed the misjudgment-specificity of rACC activation.

Previous studies have consistently implicated the ACC in error processing [23]–[28], [30], showing that the rACC activation is responsible for the affective aspects of error [42]. In addition, a recent study reported that the rACC was recruited during processing errors made by others performing the same task as the participant [31]. This suggests that the processing of observational errors also involves an affective component. Our study is in agreement with this recent work by demonstrating that the rACC is also engaged during processing errors from a third-party justice system.

The rACC has been reported to evaluate the emotional significance of errors [31], [43]–[45], participate in autonomic function [45], and predict the degree of emotional arousal [46]. Misjudgments result in a typical injustice situation, and injustice has been related with high emotional arousal [47], [48]. This high emotionally arousing character of misjudgment might result in the activation of the rACC. This notion was supported by our subjective rating data, which showed that more intense pleasure was evoked by positive misjudgments than by positive right judgments. Similarly, more intense unpleasantness was evoked by negative misjudgments than by negative correct judgments. As a result of the tight linkage between pleasure and arousal, as strongly pleasant and unpleasant stimuli also tend to be strongly arousing, it is convincing to speculate from the rating data that participants did experience more arousing emotion when they were misjudged than when they were judged rightly, independent of whether the judgment was positive or negative for them. In addition, our results showed that the activity of the rACC for positive misjudgments was correlated with that for negative misjudgments, which demonstrated that the same neural mechanism of emotional arousal processing was involved in both positive and negative misjudgments. These results suggest that humans can distinguish misjudgments from right judgments at both behavioral and neural levels. Misjudgments are therefore a form of strong emotionally arousing stimulus, regardless of whether the misjudgment is positive or negative.

With regards to evaluating the valence of judgment, subjective rating data confirmed our design for valence. It showed that positive judgments were ranked as being more pleasant than the neutral condition, while negative judgments were ranked as being more unpleasant, independent of whether the judgment was right or wrong. The valence effect was expressed in the brain activity pattern, with the striatum being activated to a larger extent by positive judgments than by negative judgments. The striatum has been reported to be engaged in reward processing, during which it is sensitively triggered by monetary gain or any events with a hedonic content [38], [49]–[53]. On the basis of previous findings and our present results, we suggest that the striatum specifically evaluates the valence of judgment and assesses positive judgments as a form of reward regardless of whether the judgment is right or wrong.

Furthermore, the result of the main effect of valence also revealed that the right posterior insula was activated to a larger extent by negative judgments than positive judgments. The insula has been demonstrated to be sensitive to salient stimuli, with the anterior insula being implicated in salient events of subjective significances, whereas the posterior insula is implicated in salient events of sensory attributes [54], [55]. In this study, participants concentrated on the red door that showed their own outcomes. Compared with the positive judgment condition, when there no feedback appeared on the red door, the negative judgment condition was manifested by a “−5” appearing on the red door, indicating that 5 Yuan was being penalized. Due to its important implication, this visual information (a red door with “−5”) became a type of salient stimulus during the whole scanning period, and thus activated the posterior insula.

Besides, the postcentral and precentral cortices in the left hemisphere were found to be more active for positive judgments than negative judgments, while these regions in the right hemisphere showed opposite activation patterns, being more active for negative judgments than for positive. As for their roles in sensory and movement processing, and their lateralization characteristics (contralateral with respect to the involved location of the body), the involvement of these areas in valence processing appears to stem from the process of subjective rating. Specifically, participants pressed “4” with their right thumb to rate positive judgments as pleasant, while they pressed “1” using their left thumb to rate judgments as unpleasant during the subjective rating stage.

In conclusion, we investigated the neural substrates of processing the judgment from a third-party justice system and demonstrated that two dissociated brain activation patterns independently evaluate the accuracy and the valence of judgments. Activation in the rACC can distinguish misjudgments from right judgments regardless of whether the misjudgment will result in loss or reward. This finding suggests that the human brain can recognize errors within misjudgments, and regards this recognition as a high emotionally arousing stimulus. The striatum are responsible for assessing the valence of judgment, regardless of whether the judgment is right or wrong, which suggests that people recognize positive judgments as hedonic information. We expect our findings regarding the involvement of the rACC in the processing of judgment accuracy to have implications in the development of a novel marker for the detection of lies.

## References

[1] Furman HP (2003) Wrongful convictions and the accuracy of the criminal justice system. Colo Lawyer 32: 11–30.

[2] Gudjonsson GH (2012) “Convicting the Innocent”: False Confessions and Correcting Injustices. New Engl Law Rev 46: 689–931.

[3] Gould JB, Leo RA (2010) One hundred years later: wongful convictions after a century of research. J Crim Law Criminol 100: 2010–2028.

[4] Leo RA (2005) Rethinking the Study of Miscarriages of Justice: Developing a Criminology of Wrongful Conviction. J Contemp Crim Justice 21: 201–223.

[5] Gudjonsson GH (2003) Psychology brings justice: the science of forensic psychology. Crim Behav Ment Health 13: 159–167. 14654868 10.1002/cbm.539

[6] Leo RA, Davis D (2009) From false confession to wrongful conviction: seven psychological processes. J Psychiat Law 38: 9–56.

[7] Ricciardelli R, Bell JG, Clow KA (2009) Student Attitudes toward Wrongful Conviction. Can J Criminol Crim Justice 51: 411–427.

[8] Swanner JK, Beike DR (2010) Incentives increase the rate of false but not true secondary confessions from informants with an allegiance to a suspect. Law Hum Behav 34: 418–428. 20107880 10.1007/s10979-009-9212-x

[9] Conti RP (1999) The psychology of false confessions. J credibil assess witn psycho 2: 14–36.

[10] Spence SA, Farrow TFD, Herford AE, Wilkinson ID, Zheng Y, et al (2001) Behavioural and functional anatomical correlates of deception in humans. Neuroreport 12: 2849–2853. 11588589 10.1097/00001756-200109170-00019

[11] Langleben DD, Schroeder L, Maldjian JA, Gur RC, McDonald S, et al (2002) Brain activity during simulated deception: an event-related functional magnetic resonance study. NeuroImage 15: 727–732. 11848716 10.1006/nimg.2001.1003

[12] Kozel FA, Padgett TM, George MS (2004) A replication study of the neural correlates of deception. Behav Neurosci 118: 852–856. 15301611 10.1037/0735-7044.118.4.852

[13] Kozel FA, Revell LJ, Lorberbaum JP, Shastri A, Elhai JD, et al (2004) A pilot study of functional magnetic resonance imaging brain correlates of deception in healthy young men. J Neuropsychiatry Clin Neurosci 16: 295–305. 15377736 10.1176/jnp.16.3.295

[14] Davatzikos C, Ruparel K, Fan Y, Shen DG, Acharyya M, et al (2005) Classifying spatial patterns of brain activity with machine learning methods: application to lie detection. NeuroImage 28: 663–668. 16169252 10.1016/j.neuroimage.2005.08.009

[15] Kozel FA, Johnson KA, Mu Q, Grenesko EL, Laken SJ, et al (2005) Detecting deception using functional magnetic resonance imaging. Biol Psychiatry 58: 605–613. 16185668 10.1016/j.biopsych.2005.07.040

[16] Kozel FA, Laken SJ, Johnson KA, Boren B, Mapes KS, et al (2009) Replication of functional MRI detection of deception. Open Forensic Sci J 2: 6–11. 19844599 10.2174/1874402800902010006 PMC2763380

[17] Hakun JG, Seelig D, Ruparel K, Loughead JW, Busch E, et al (2008) fMRI investigation of the cognitive structure of the Concealed Information Test. Neurocase 14: 59–67. 18569732 10.1080/13554790801992792

[18] Kozel FA, Johnson KA, Grenesko EL, Laken SJ, Kose S, et al (2009) Functional MRI Detection of Deception After Committing a Mock Sabotage Crime. J Forensic Sci 54: 220–231. 19067772 10.1111/j.1556-4029.2008.00927.x PMC2735094

[19] Langleben DD (2008) Detection of deception with fMRI: Are we there yet? Legal Criminol Psych 13: 1–9.

[20] Wolpe PR, Foster KR, Langleben DD (2005) Emerging neurotechnologies for lie-detection: promises and perils. Am J Bioeth 5: 39–49. 16036700 10.1080/15265160590923367

[21] Spence SA (2004) The deceptive brain. J R Soc Med 97: 6–9. 14702355 10.1258/jrsm.97.1.6 PMC1079256

[22] Sip KE, Roepstorff A, McGregor W, Frith CD (2008) Detecting deception: the scope and limits. Trends Cogn Sci 12: 48–53. 18178516 10.1016/j.tics.2007.11.008

[23] Falkenstein M, Hohnsbein J, Hoormann J, Blanke L (1991) Effects of crossmodal divided attention on late ERP components. II. Error processing in choice reaction tasks. Electroencephalogr Clin Neurophysiol 78: 447–455. 1712280 10.1016/0013-4694(91)90062-9

[24] Gehring WJ, Gratton G, Coles MG, Donchin E (1992) Probability effects on stimulus evaluation and response processes. J Exp Psychol Hum Percept Perform 18: 198–216. 1532188 10.1037/0096-1523.18.1.198

[25] Kiehl KA, Liddle PF, Hopfinger JB (2000) Error processing and the rostral anterior cingulate: an event-related fMRI study. Psychophysiology 37: 216–223. 10731771

[26] Swick D, Turken AU (2002) Dissociation between conflict detection and error monitoring in the human anterior cingulate cortex. Proc Natl Acad Sci U S A 99: 16354–16359. 12456882 10.1073/pnas.252521499 PMC138615

[27] Hogan AM, Vargha-Khadem F, Saunders DE, Kirkham FJ, Baldeweg T (2006) Impact of frontal white matter lesions on performance monitoring: ERP evidence for cortical disconnection. Brain 129: 2177–2188. 16815874 10.1093/brain/awl160

[28] Ullsperger M, von Cramon DY (2006) The role of intact frontostriatal circuits in error processing. J Cogn Neurosci 18: 651–664. 16768367 10.1162/jocn.2006.18.4.651

[29] Jocham G, Neumann J, Klein TA, Danielmeier C, Ullsperger M (2009) Adaptive coding of action values in the human rostral cingulate zone. J Neurosci 29: 7489–7496. 19515916 10.1523/JNEUROSCI.0349-09.2009 PMC2734938

[30] Nee DE, Kastner S, Brown JW (2011) Functional heterogeneity of conflict, error, task-switching, and unexpectedness effects within medial prefrontal cortex. NeuroImage 54: 528–540. 20728547 10.1016/j.neuroimage.2010.08.027 PMC2962721

[31] Shane MS, Stevens M, Harenski CL, Kiehl KA (2008) Neural correlates of the processing of another’s mistakes: a possible underpinning for social and observational learning. NeuroImage 42: 450–459. 18534868 10.1016/j.neuroimage.2007.12.067 PMC2593145

[32] de Bruijn ERA, de Lange FP, von Cramon DY, Ullsperger M (2009) When errors are rewarding. J Neurosci 29: 12183–12186. 19793976 10.1523/JNEUROSCI.1751-09.2009 PMC6666159

[33] Yoshida K, Saito N, Iriki A, Isoda M (2012) Social error monitoring in macaque frontal cortex. Nat Neurosci 15: 1307–1312. 22864610 10.1038/nn.3180

[34] Klein TA, Neumann J, Reuter M, Hennig J, von Cramon DY, et al (2007) Genetically determined differences in learning from errors. Science 318: 1642–1645. 18063800 10.1126/science.1145044

[35] Knutson B, Cooper JC (2005) Functional magnetic resonance imaging of reward prediction. Curr Opin Neurol 18: 411–417. 16003117 10.1097/01.wco.0000173463.24758.f6

[36] O’Doherty JP, Hampton A, Kim H (2007) Model-based fMRI and its application to reward learning and decision making. Ann NY Acad Sci 1104: 35–53. 17416921 10.1196/annals.1390.022

[37] Pagnoni G, Zink CF, Montague PR, Berns GS (2002) Activity in human ventral striatum locked to errors of reward prediction. Nat Neurosci 5: 97–98. 11802175 10.1038/nn802

[38] Takahashi H, Kato M, Matsuura M, Mobbs D, Suhara T, et al (2009) When your gain is my pain and your pain is my gain: neural correlates of envy and schadenfreude. Science 323: 937–939. 19213918 10.1126/science.1165604

[39] Ullsperger M, von Cramon DY (2003) Error monitoring using external feedback: specific roles of the habenular complex, the reward system, and the cingulate motor area revealed by functional magnetic resonance imaging. J Neurosci 23: 4308–4314. 12764119 10.1523/JNEUROSCI.23-10-04308.2003 PMC6741115

[40] Declaration of Helsinki (BMJ 1991; 302: 1194).

[41] O’Doherty JP (2004) Reward representations and reward-related learning in the human brain: insights from neuroimaging. Curr Opin Neurobiol 14: 769–776. 15582382 10.1016/j.conb.2004.10.016

[42] Bush G, Luu P, Posner MI (2000) Cognitive and emotional influences in anterior cingulate cortex. Trends Cogn Sci 4: 215–222. 10827444 10.1016/s1364-6613(00)01483-2

[43] Taylor SF, Martis B, Fitzgerald KD, Welsh RC, Abelson JL, et al (2006) Medial frontal cortex activity and loss-related responses to errors. J Neurosci 26: 4063–4070. 16611823 10.1523/JNEUROSCI.4709-05.2006 PMC6673891

[44] Laurens KR, Ngan ETC, Bates AT, Kiehl KA, Liddle PF (2003) Rostral anterior cingulate cortex dysfunction during error processing in schizophrenia. Brain 126: 610–622. 12566282 10.1093/brain/awg056

[45] Phillips ML, Drevets WC, Rauch SL, Lane R (2003) Neurobiology of emotion perception I: The neural basis of normal emotion perception. Biol Psychiatry 54: 504–514. 12946879 10.1016/s0006-3223(03)00168-9

[46] Critchley HD, Tang J, Glaser D, Butterworth B, Dolan RJ (2005) Anterior cingulate activity during error and autonomic response. NeuroImage 27: 885–895. 15996878 10.1016/j.neuroimage.2005.05.047

[47] van ’t Wout M, Kahn RS, Sanfey AG, Aleman A (2006) Affective state and decision-making in the Ultimatum Game. Exp Brain Res 169: 564–568. 16489438 10.1007/s00221-006-0346-5

[48] De Cremer D (2007) Advances in the psychology of justice and affect. CharlotteNC: Information Age Publishing. viii, 292 p.

[49] Takahashi H, Matsuura M, Koeda M, Yahata N, Suhara T, et al (2008) Brain activations during judgments of positive self-conscious emotion and positive basic emotion: pride and joy. Cereb Cortex 18: 898–903. 17638925 10.1093/cercor/bhm120

[50] Balleine BW, Delgado MR, Hikosaka O (2007) The role of the dorsal striatum in reward and decision-making. J Neurosci 27: 8161–8165. 17670959 10.1523/JNEUROSCI.1554-07.2007 PMC6673072

[51] Delgado MR, Nystrom LE, Fissell C, Noll DC, Fiez JA (2000) Tracking the hemodynamic responses to reward and punishment in the striatum. J Neurophysiol 84: 3072–3077. 11110834 10.1152/jn.2000.84.6.3072

[52] Elliott R, Newman JL, Longe OA, Deakin JFW (2003) Differential response patterns in the striatum and orbitofrontal cortex to financial reward in humans: a parametric functional magnetic resonance imaging study. J Neurosci 23: 303–307. 12514228 10.1523/JNEUROSCI.23-01-00303.2003 PMC6742125

[53] Mobbs D, Greicius MD, Abdel-Azim E, Menon V, Reiss AL (2003) Humor modulates the mesolimbic reward centers. Neuron 40: 1041–1048. 14659102 10.1016/s0896-6273(03)00751-7

[54] Menon V, Uddin LQ (2010) Saliency, switching, attention and control: a network model of insula function. Brain Struct Funct 214: 655–667. 20512370 10.1007/s00429-010-0262-0 PMC2899886

[55] Craig A (2009) How do you feel–now? The anterior insula and human awareness. Nat Rev Neurosci 10: 59–70. 19096369 10.1038/nrn2555

